# Supplementary material for: Informing antimicrobial stewardship with explainable AI
Source: PLOS Digit Health. 2023 Jan 5;2(1):e0000162. doi: 10.1371/journal.pdig.0000162 (PMC9931350; doi:10.1371/journal.pdig.0000162)
Supplement: S1 Text — (PDF) [file pdig.0000162.s001.pdf]

Supporting information – S1 Text.
Informing antimicrobial stewardship with explainable AI

Massimo Cavallaro[1,2,*], Ed Moran[3], Benjamin Collyer[4], Noel D. McCarthy[5,6], Christopher Green[7,8], Matt J. Keeling[1,2]

1 School of Life Sciences and Mathematics Institute, University of Warwick, Coventry, United Kingdom
2 The Zeeman Institute for Systems Biology & Infectious Disease Epidemiology Research, University of Warwick, Coventry, United Kingdom
3 Department of Infectious Disease, North Bristol NHS Trust, Bristol, United Kingdom
4 Faculty of Medicine, School of Public Health, Imperial College London, London, United Kingdom
5 Institute of Population Health, Trinity College Dublin, University of Dublin, Dublin, Ireland
6 Warwick Medical School, University of Warwick, Coventry, United Kingdom
7 Institute of Microbiology & Infection, University of Birmingham, Birmingham, United Kingdom
8 Department of Infectious Diseases & Tropical Medicine, University Hospitals Birmingham NHS Foundation Trust

* m.cavallaro@warwick.ac.uk

Supporting information text, including: Fig A and Tables A, B, C, D, and E.

Abbreviation	Admission method
A&E	Accident and emergency or dental casualty department of the Health Care Provider
A&E other provider	Accident and emergency Department of another provider where the patient had not been admitted
Ante partum	Admitted ante partum
Bed bureau	Bed bureau
Booked	Booked
Consultant clinic	Consultant Clinic, of this or another Health Care Provider
GP	General Practitioner
Other emergency	Other emergency admission
Planned	Planned
Transfer (emergency)	Transfer of an admitted patient from another Hospital Provider in an emergency
Transfer (non-emerg.)	Transfer of an admitted patient from other Hospital Provider other than in an emergency
Waiting list	Waiting list
Other	Other means

Table A. Admission methods included in the study and their abbreviations.

Consultant specialty
Accident & emergency
Cardiology
Clinical haematology
Clinical oncology (previously radiotherapy)
Dermatology
Ent
Gastroenterology
General medicine
General surgery
Geriatric medicine
Gynaecology
Infectious diseases
Medical oncology
Nephrology
Obstetrics
Plastic surgery
Respiratory medicine (also known as thoracic medicine)
Rheumatology
Trauma & orthopaedics
Urology
Other

Table B. List of consultant specialties included in the study.

Hyper-parameter name	AUG	CIP	MEM	TAZ
`gamma`	7.82	16.02	14.17	10.26
`learning_rate`	0.06	0.10	0.20	0.04
`max_delta_step`	4.13	9.45	8.33	4.91
`max_depth`	10	10	10	8
`min_child_weight`	3.35	3.16	4.63	3.71
`subsample`	0.78	0.88	0.77	0.59
`scale_pos_weight`	-	1.76	3.41	1.78

Table C. Xgboost hyper-parameters selected by means of optimisation of c-statistics using 5-fold cross-validation over the training set (70% of all observations) with constant L1-regularisation parameter $\alpha = 0.5$ for each outcome.

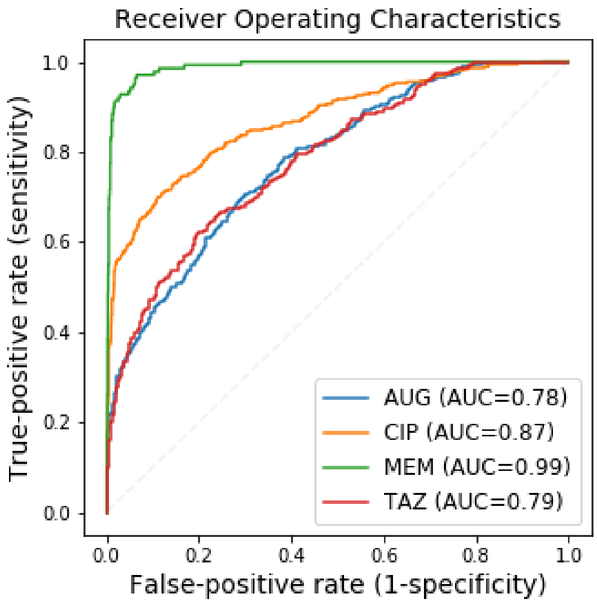

Fig A. Receiver operating characteristic curves for resistance prediction computed over the test set (30% of all observations).

True prescr.	Physician outcome	AI predic.	AI prescr.	True outcome	Physician mism.	AI mism.
1	S	0.1	1	S	0	0
0	S	0.1	1	S	0	0
0	?	0.2	0	S	-	-
1	S	0.2	1	R	1	1
1	S	0.3	1	R	1	1
1	S	0.4	1	S	0	0
1	S	0.5	0	R	1	-
0	?	0.6	0	S	-	-
0	?	0.7	0	R	-	-
0	?	0.9	0	R	-	-

Table D. Alternative comparison between true and AI assisted prescriptions (strategy 2). For a given antimicrobial drug and inpatient, data contains prescription records (first column, 1 if the drug was prescribed during admission, 0 otherwise), and later culture test (fifth column – "True outcome", S or R if the isolate was found susceptible or resistant to the drug, respectively). Drug treatment corresponds to a physician's prediction (second column – a physician prescribes a drug if they believe that isolates are susceptible to the drug, while nothing can be said if a drug is not prescribed). By comparing true outcomes to physician predictions, the number of matches and mismatches can be computed (here 3 matches and 3 mismatches – sixth column). An AI system such as the GBDT model returns the probability that an isolate is found resistant (third column – "AI predic."). To leverage these predictions, AI prescriptions (fourth column) are given to inpatients from top to bottom (lowest to highest probability of AMR) until the number of matches (marked by zeros in 7th column) equals that from true prescription. As a result, the number of mismatches obtained with this AI strategy (2 in the 7th column) is smaller than the observed number of matches (in the 6th column). Prescr.=prescription, predic.=prediction, mism.=mismatch.

Outcome	Factor	Σ	Gain	I	95% CI	log OR	95% CI
AUG	Isolate susceptible to AUG previously found	0.63	20.90	−0.46	−0.47, −0.44		
	Isolate resistant to AUG previously found	0.40	58.64	0.58	0.56, 0.60		
	Admi. date	0.15	6.53	−0.00	−0.00, −0.00		
	AUG prescribed in past admissions	0.14	8.65	0.18	0.17, 0.19	0.46	0.31, 0.62
	KPNE	0.13	8.31	−0.62	−0.62, −0.61	−0.94	−1.22, −0.66
	Isolate susceptible to CIP previously found	0.11	10.65	0.07	0.06, 0.07		
	Total time in hosp.	0.10	7.04	0.00	0.00, 0.00		
	Isolate resistant previously found	0.09	8.74	0.26	0.25, 0.26	0.77	0.60, 0.95
	Early test	0.08	6.34	−0.14	−0.14, −0.14	−0.31	−0.47, −0.15
	Isolate susceptible to ETP previously found	0.08	9.45	0.04	0.03, 0.04		
	Septicaemia	0.07	7.33	0.22	0.22, 0.23	0.49	0.29, 0.69
	Frac. time in hosp.	0.06	6.63	0.06	0.06, 0.07		
	Isolate susceptible to CAZ previously found	0.05	8.65	−0.03	−0.03, −0.02		
	AUG early prescribed	0.05	4.91	0.12	0.12, 0.12	0.08	−0.11, 0.27
	Isolate susceptible to TAZ previously found	0.05	7.08	−0.05	−0.05, −0.05		
	ETP prescribed in current admission	0.04	7.12	0.24	0.23, 0.24	0.53	0.27, 0.80
CIP	Isolate resistant to CIP previously found	0.83	283.89	0.07	0.07, 0.07		
	Isolate susceptible to CIP previously found	0.77	24.28	−0.19	−0.20, −0.18		
	Isolate susceptible to MEM previously found	0.24	24.39	0.06	0.06, 0.07		
	Admi. date	0.18	11.36	−0.00	−0.00, −0.00		
	Total time in hosp.	0.13	13.81	0.00	0.00, 0.00		
	ECOL	0.10	12.62	0.06	0.06, 0.07	−0.66	−0.81, −0.50
	Cystic fibrosis	0.10	18.44	0.54	0.53, 0.54	2.82	2.55, 3.09
	Septicaemia	0.09	13.11	0.27	0.27, 0.28	0.25	0.05, 0.45
	CIP prescribed in past admissions	0.08	18.61	0.41	0.41, 0.42	1.78	1.55, 2.01
	AUG prescribed in current admission	0.06	13.67	−0.08	−0.08, −0.07	−1.03	−1.20, −0.86
	MEM prescribed in current admission	0.05	11.61	0.29	0.29, 0.30	1.18	0.95, 1.41
MEM	ECOL	1.22	32.63	−1.52	−1.54, −1.51	−6.31	−7.91, −4.70
	Isolate resistant to MEM previously found	0.76	427.01	0.05	0.05, 0.06		
	PAER	0.71	58.13	0.94	0.90, 0.98	5.47	4.68, 6.25
	Isolate susceptible to MEM previously found	0.23	10.05	−0.03	−0.04, −0.03		
	Cystic fibrosis	0.22	590.01	0.59	0.57, 0.61	5.14	4.76, 5.51
	Admi. Date	0.19	13.02	0.00	−0.00, 0.00		
	Age	0.18	15.13	−0.00	−0.00, −0.00		
	MEM prescribed in past admissions	0.18	21.71	0.22	0.21, 0.24	3.64	3.33, 3.95
	Consultant specialty: General medicine	0.12	11.62	−0.43	−0.43, −0.42	−3.52	−4.76, −2.27
	Frac. time in hosp.	0.12	11.40	0.12	0.11, 0.13		

Table E. Importance of the top 75% factors ranked by their absolute sum Σ of Shapley values for antibiotic resistance prediction (continues in Table F, next page).

Outcome	Factor	Σ	Gain	I	95% CI	log OR	95% CI
TAZ	Isolate susceptible to TAZ previously found	0.61	17.20	−0.05	−0.06, −0.05		
	Isolate resistant to TAZ previously found	0.52	53.24	0.08	0.08, 0.08		
	Total time in hosp.	0.16	10.99	0.00	0.00, 0.00		
	Isolate susceptible to AUG previously found	0.14	12.25	−0.13	−0.13, −0.13		
	Admi. date	0.14	9.44	0.00	0.00, 0.00		
	Isolate resistant previously found	0.11	15.69	0.22	0.22, 0.23	0.96	0.79, 1.13
	Age	0.09	9.20	−0.00	−0.00, −0.00		
	Isolate susceptible to MEM previously found	0.09	14.61	0.02	0.02, 0.02		
	ECOL	0.06	8.82	−0.06	−0.06, −0.05	−0.68	−0.85, −0.51
	Frac. time in hosp.	0.06	9.05	0.03	0.02, 0.04		
	AUG prescribed in past admissions	0.05	8.67	0.06	0.06, 0.06	0.13	−0.04, 0.30
	Isolate susceptible to MEM previously found	0.05	11.37	0.01	0.01, 0.01		
	AUG early prescribed	0.05	7.91	0.13	0.12, 0.13	−0.05	−0.27, 0.17
	Length of stay	0.05	9.28	0.00	−0.00, 0.00		
	TAZ prescribed in past admissions	0.04	10.12	0.10	0.10, 0.11	0.55	0.36, 0.73
	Isolate resistant to CIP previously found	0.04	7.87	0.00	0.00, 0.00		
	# of admissions	0.04	7.30	−0.00	−0.00, −0.00		
	Isolate susceptible to CAZ previously found	0.03	10.06	0.00	0.00, 0.00		
	Isolate susceptible to GT previously found	0.03	9.21	0.01	0.00, 0.01		
	Septicaemia	0.03	8.35	0.09	0.09, 0.09	0.15	−0.07, 0.36

Table F. Importance of the top 75% factors ranked by their absolute sum Σ of Shapley values for antibiotic resistance prediction (cont'ed from Table E, previous page). Σ is associated in rank order with the gain global score (Spearman's $\rho = 0.7, 0.7, 0.3, 0.7$ for AUG, CIP, MEM, TAZ, respectively, $P < 0.01$); higher values of these metrics imply a feature is more important for generating an predicted outcome compared to another feature. The slope index I, in contrast, quantifies the direction of the feature's contribution to the outcome, can be positive or negative, and, for binary factors, is correlated with the logarithm of the ratio (log OR) of the odds of AMR if exposed and the odds if not exposed (Spearman's $\rho = 0.9, 0.7, 0.8, 0.6$ for AUG, CIP, MEM, TAZ, respectively, $P < 0.0001$).
