# Supplementary figures and images for: Informing antimicrobial stewardship with explainable AI
Source: PLOS Digit Health. 2023 Jan 5;2(1):e0000162. doi: 10.1371/journal.pdig.0000162 (PMC9931350; doi:10.1371/journal.pdig.0000162)

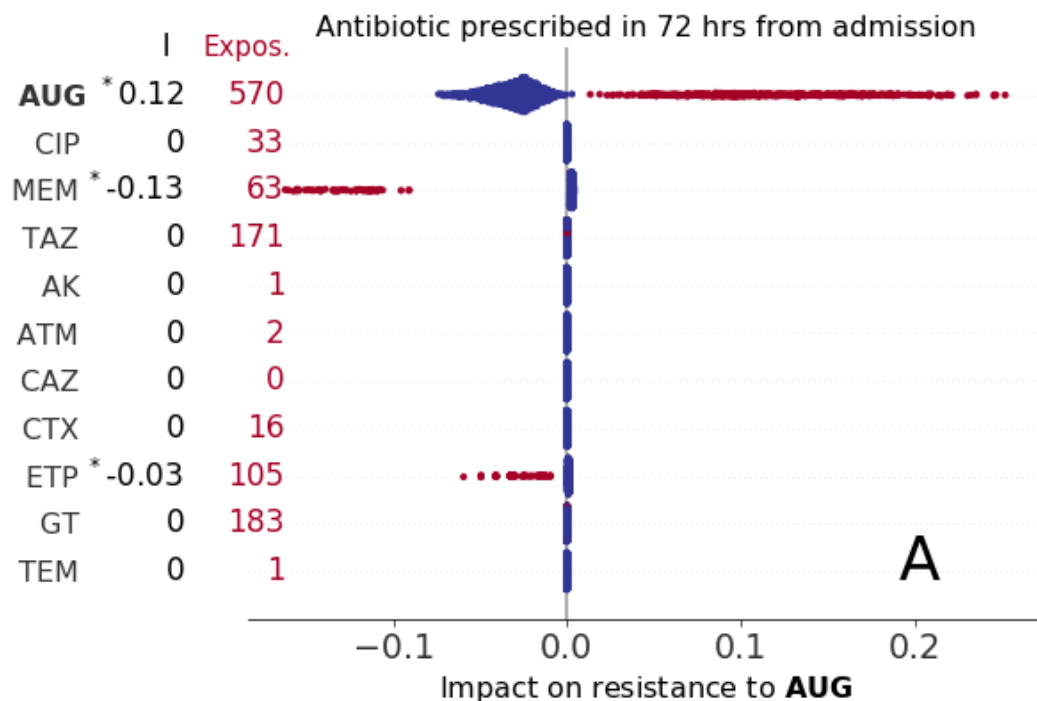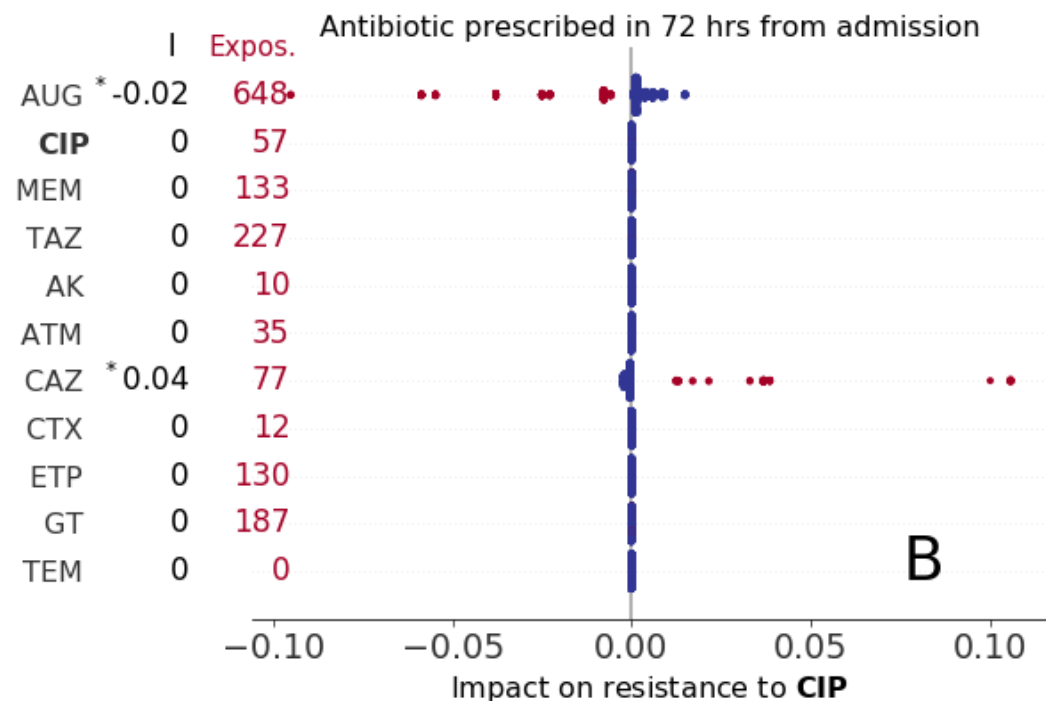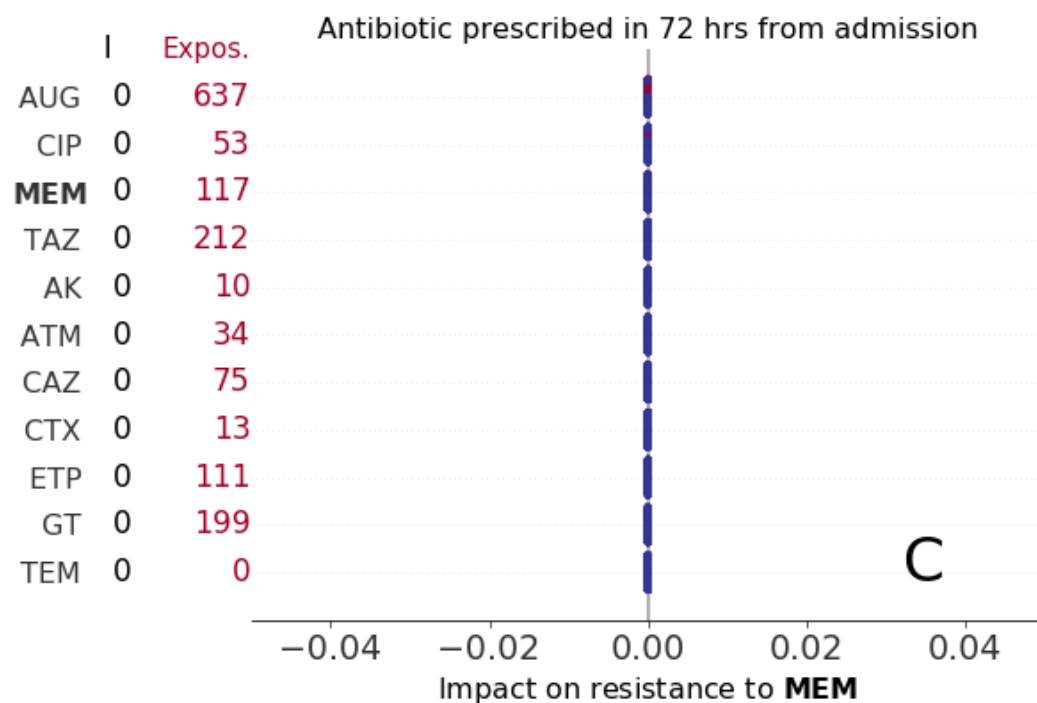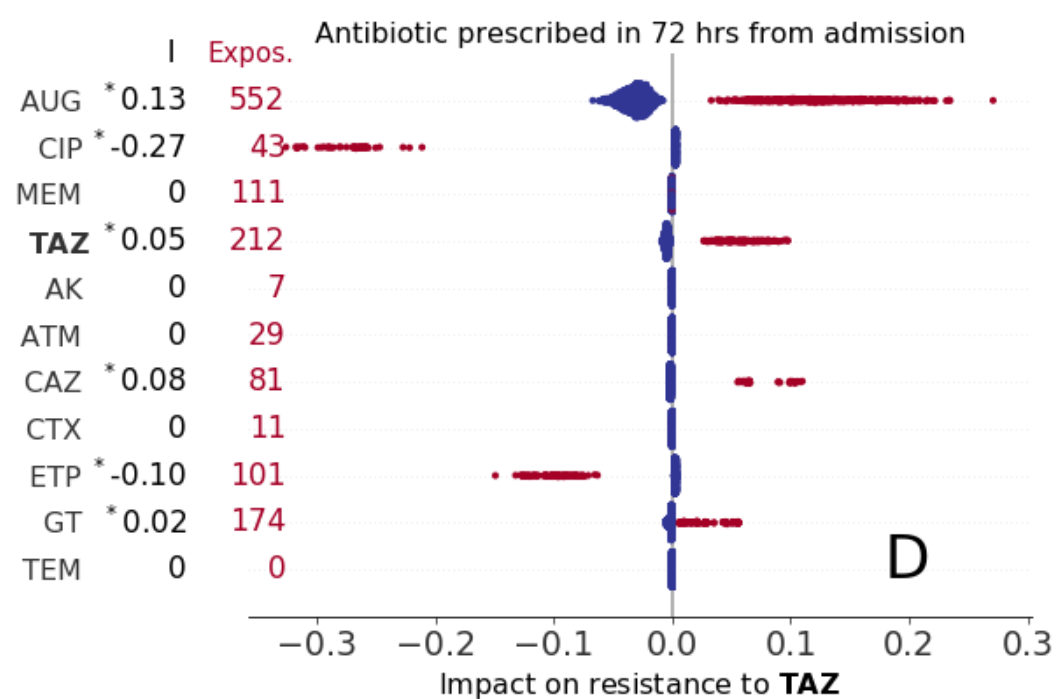

Supplement: S1 Fig — Each marker represents an admission event, with colour red indicating exposure to the treatment during the first 72 hours and total exposure reported on the left for each drug. Keys as in Fig 2. (PDF) [file pdig.0000162.s002.pdf]

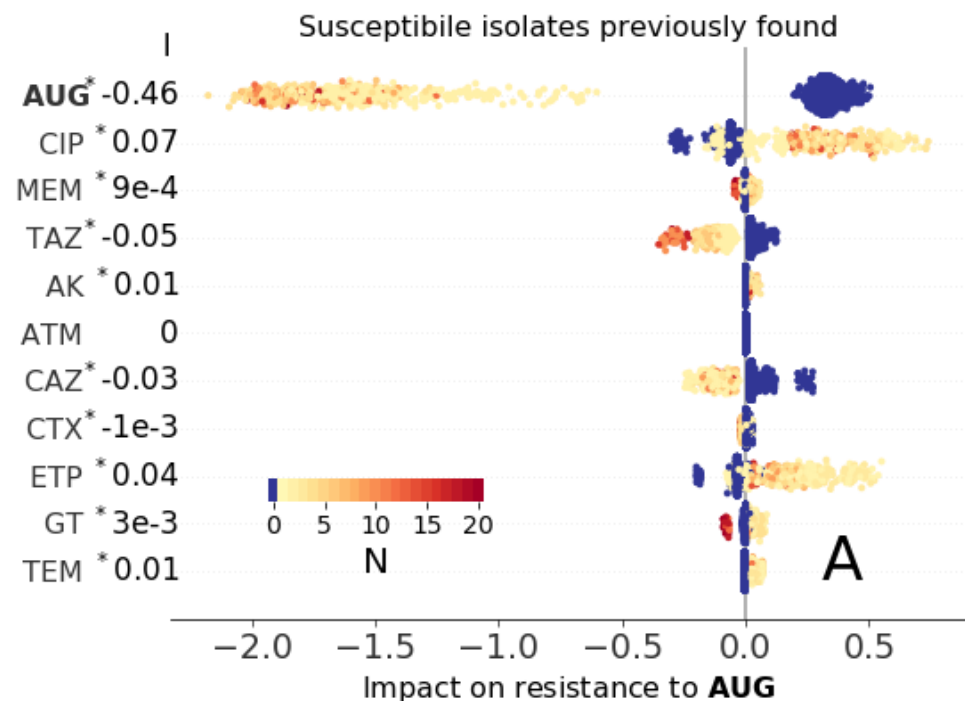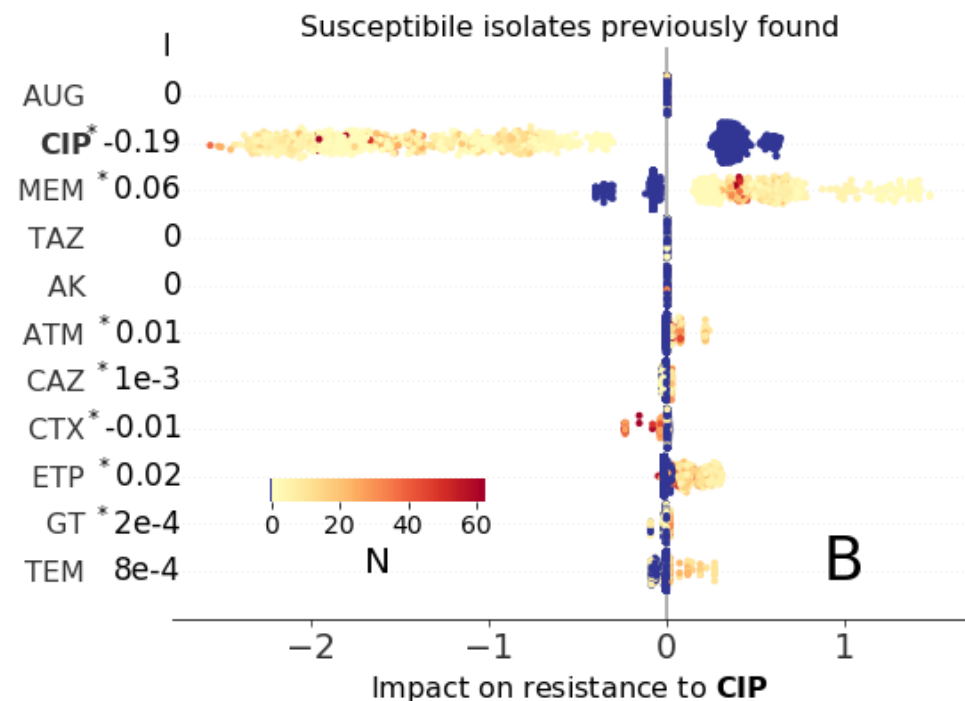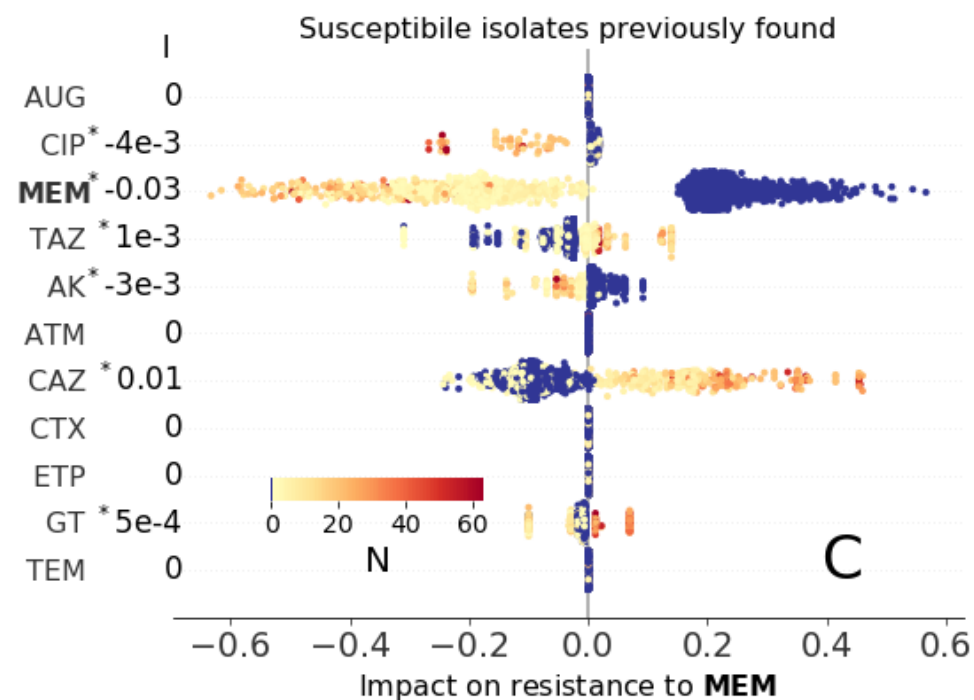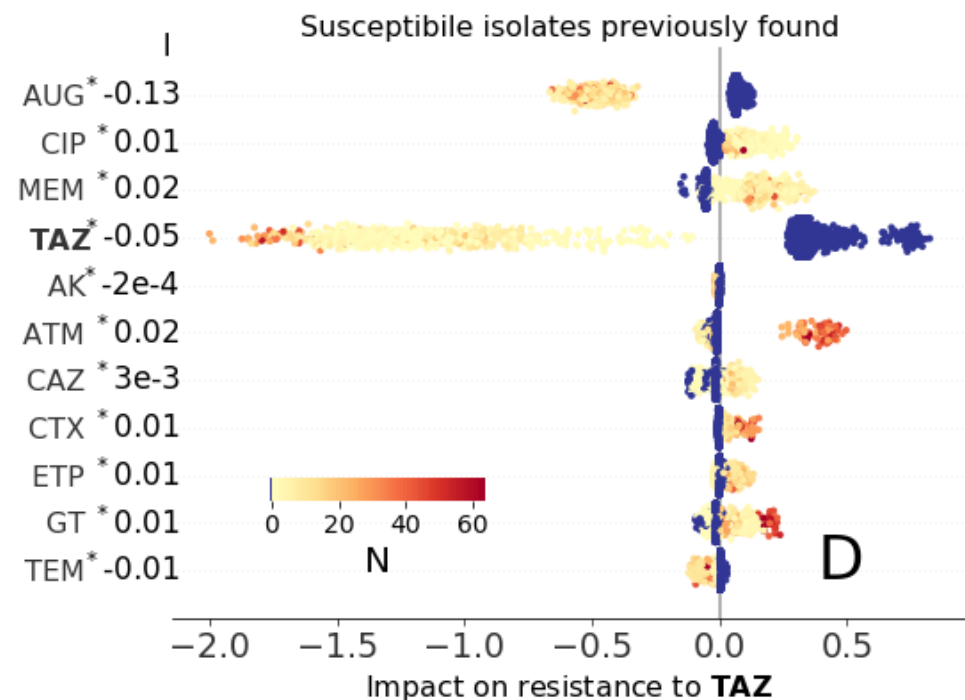

Supplement: S2 Fig — Blue marker color indicates no susceptible isolates were previously found, while light orange to dark red colors correspond to increasing number N of times an isolate was found susceptible in past admissions (inset colormaps). All other keys are as in Fig 4. (PDF) [file pdig.0000162.s003.pdf]

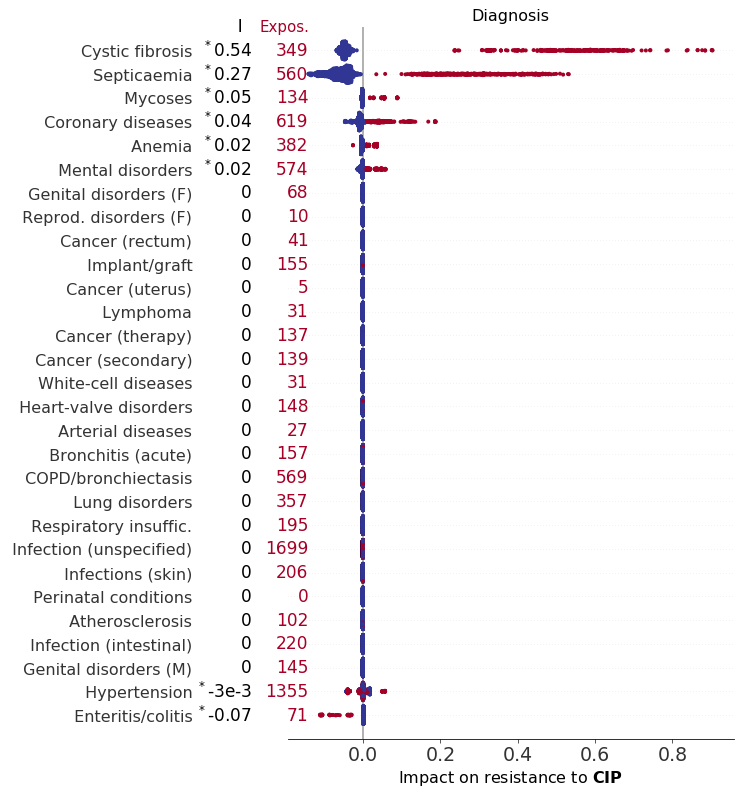

Supplement: S3 Fig — Factors are ranked by index I (top to bottom, values of I reported on the left), which measures the direction and strength of a factor’s association with outcome (see also Table E in S1 Text, asterisk (*) indicates statistical significance, P<0.01). Each marker represents an admission event, horizontal coordinates representing Shapley values, with colour red indicating presence of morbidity, and total number of diagnosed inpatients reported on the left for each disease (Expos.). (PNG) [file pdig.0000162.s004.png]

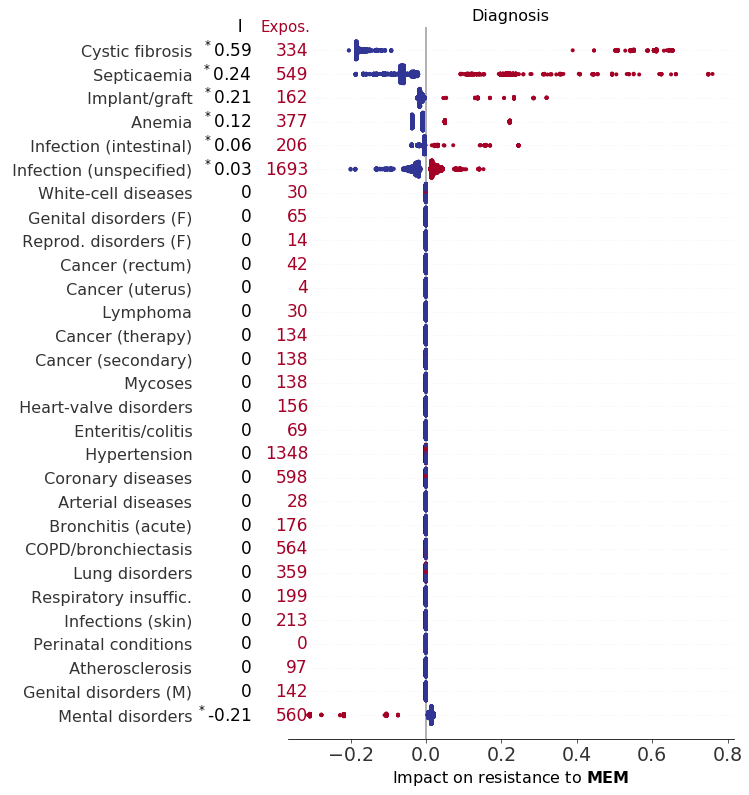

Supplement: S4 Fig — Keys as in S3 Fig. (PNG) [file pdig.0000162.s005.png]

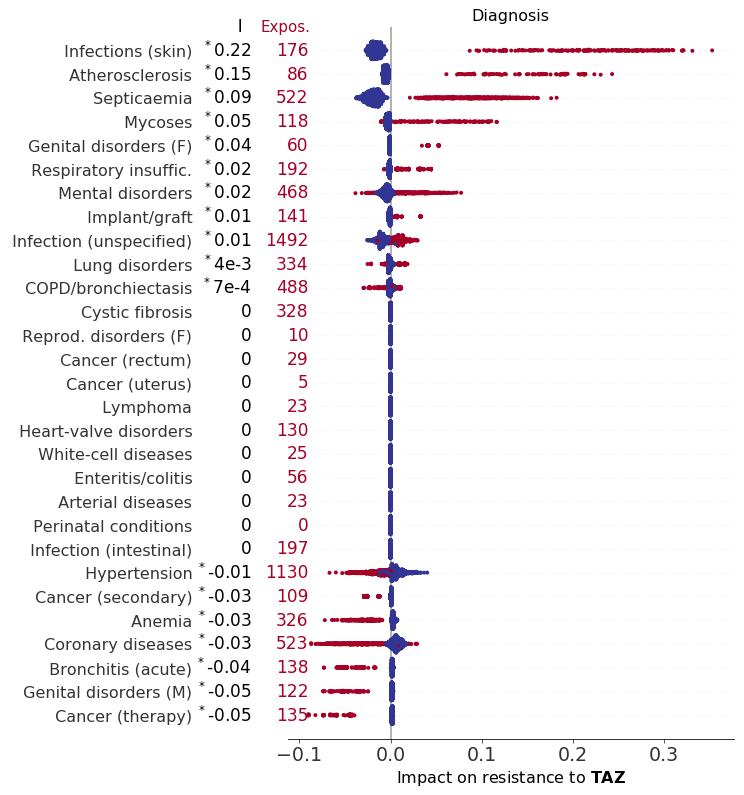

Supplement: S5 Fig — Keys as in S3 Fig. (PNG) [file pdig.0000162.s006.png]

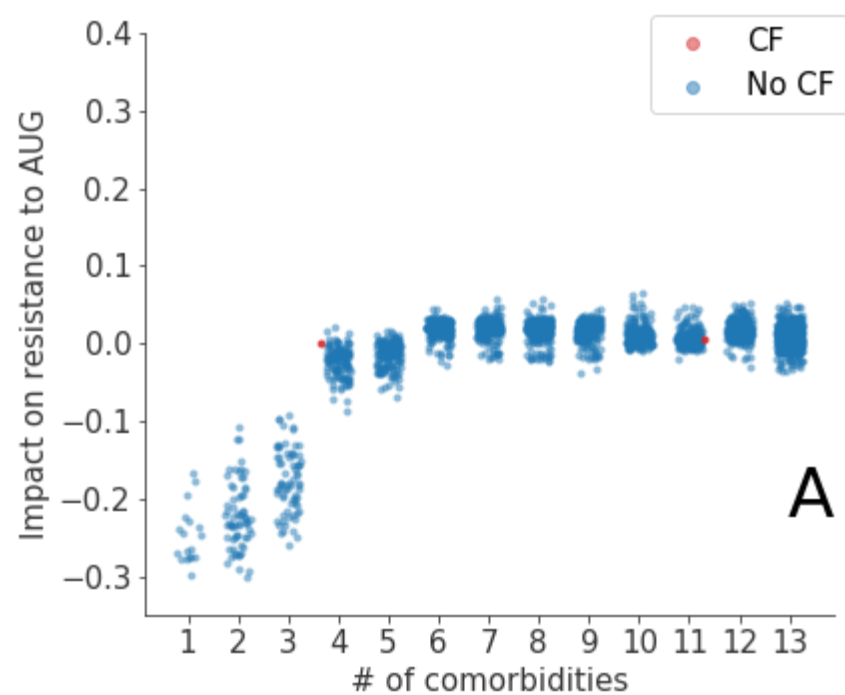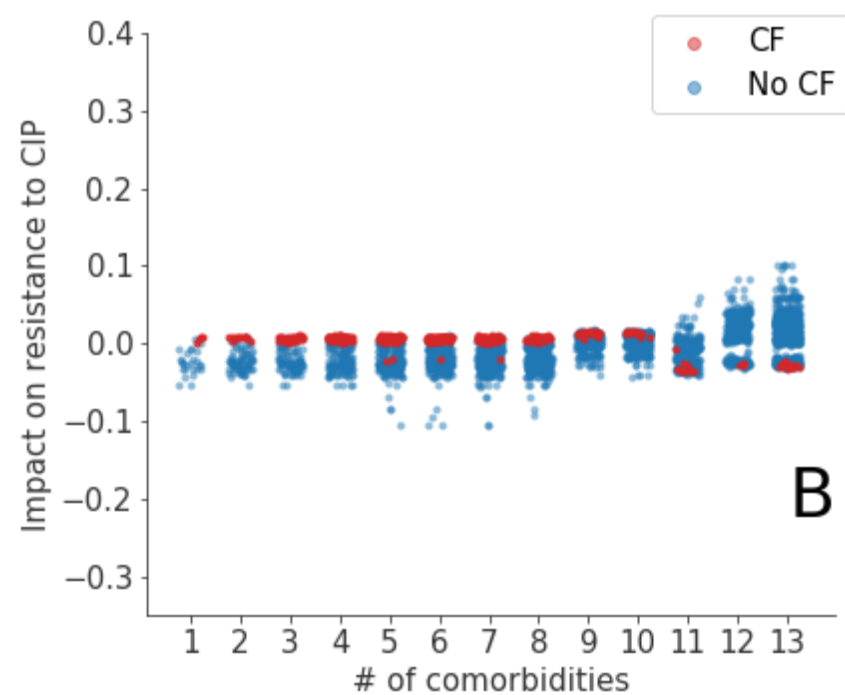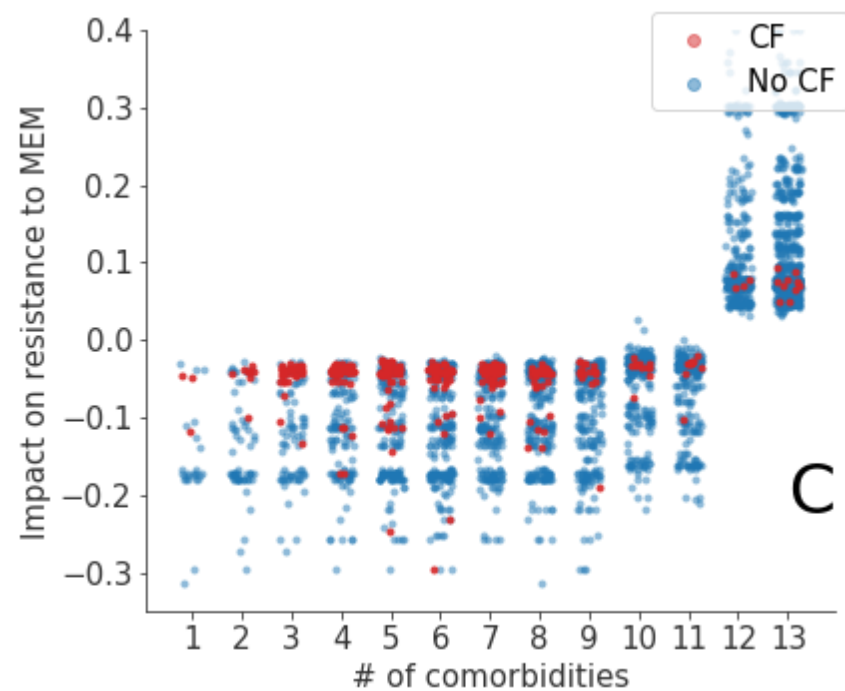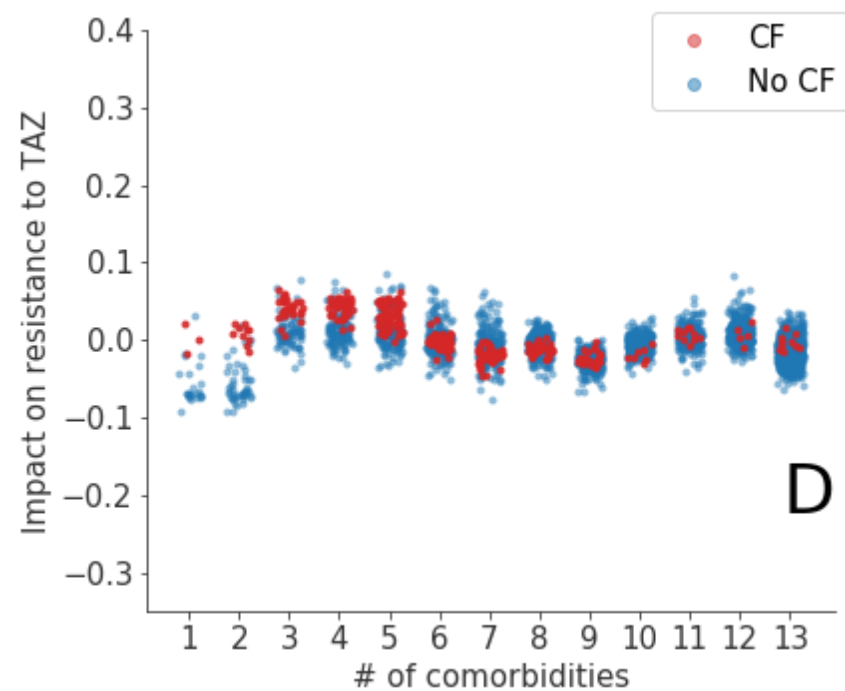

Supplement: S6 Fig — Red and blue markers correspond to cystic fibrosis (CF) and non-CF inpatients, respectively. Jitter along x axis introduced to avoid marker overlap. (PDF) [file pdig.0000162.s007.pdf]

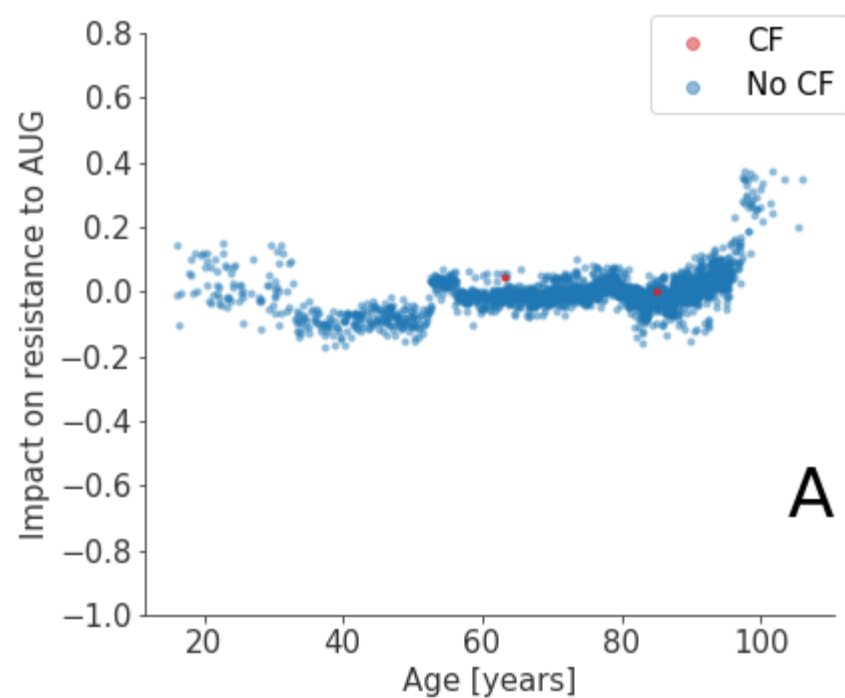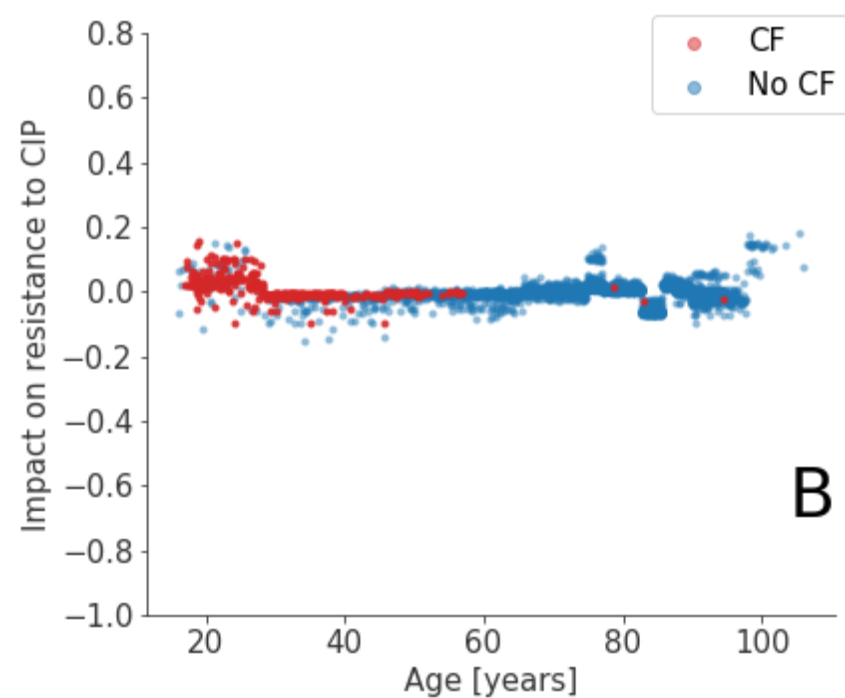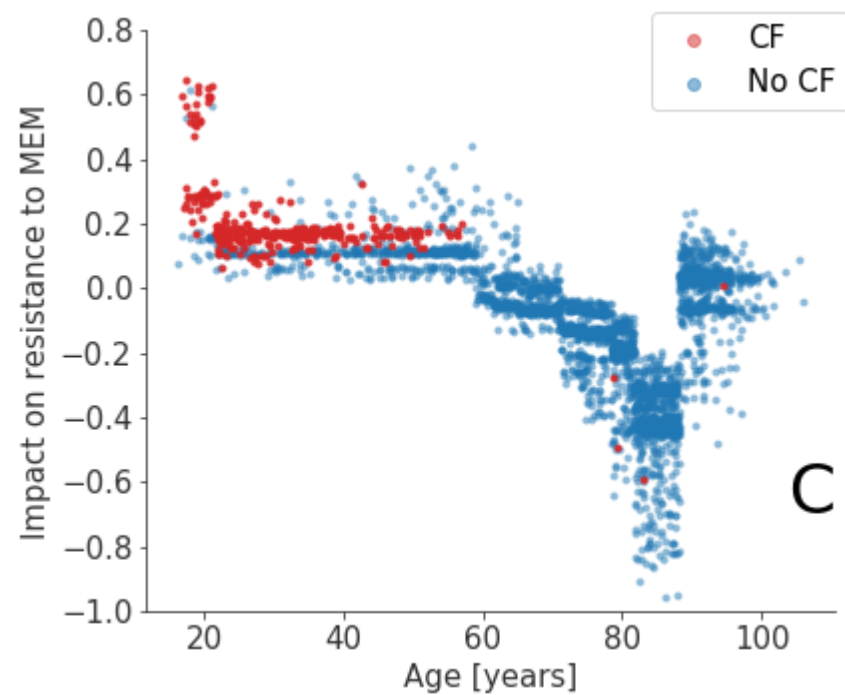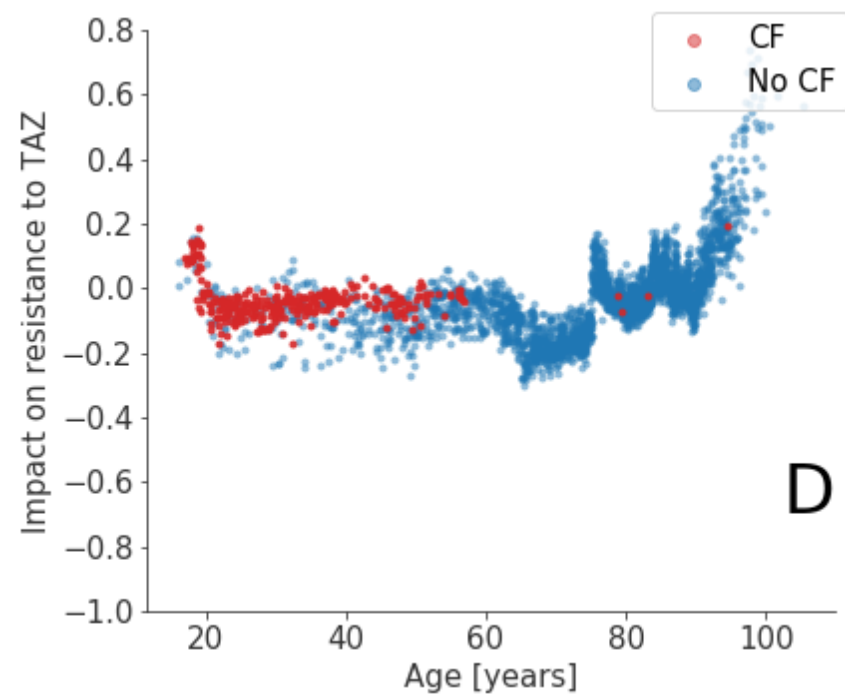

Supplement: S7 Fig — Red and blue markers correspond to cystic fibrosis (CF) and non-CF inpatients, respectively. CF inpatients are younger on average (age<60) but the impact of age does not appear substantially different than non-CF. (PDF) [file pdig.0000162.s008.pdf]

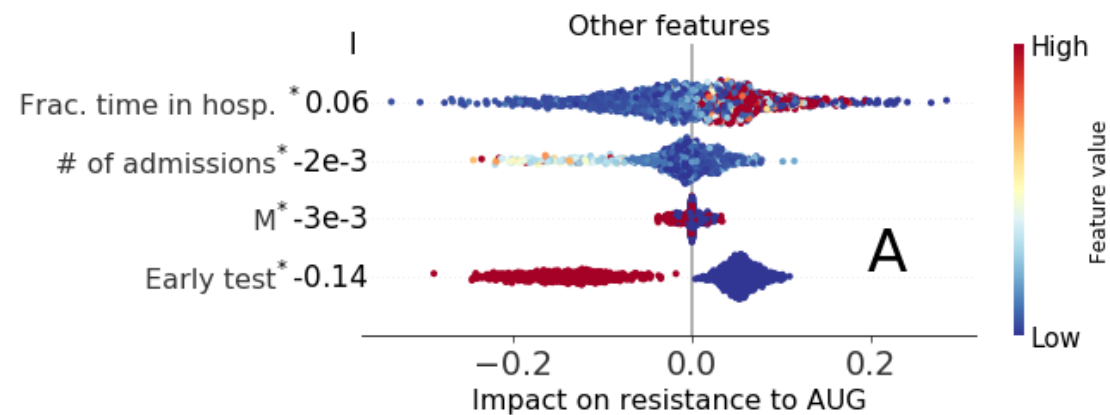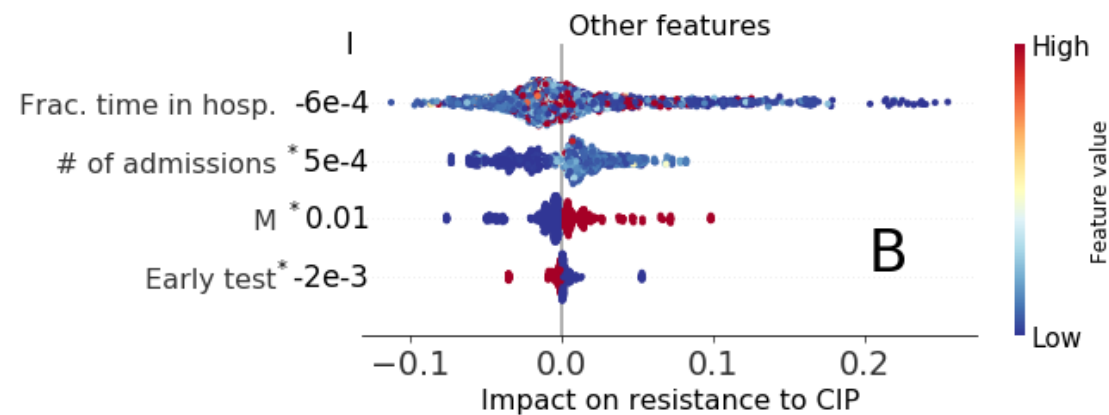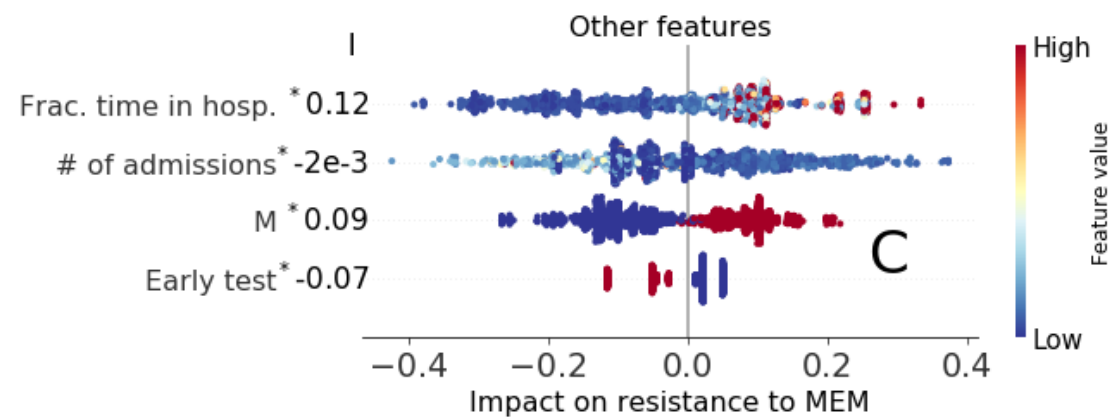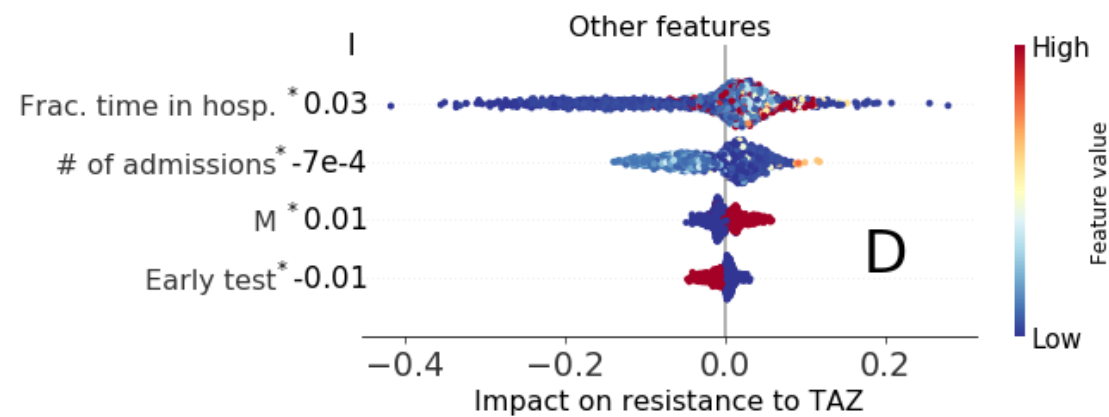

Supplement: S8 Fig — Exposures of male sex and early testing are (1008, 957), (1371, 1278), (1378, 1239), and (1255, 1082), for AUG, CIP, MEM, and TAZ, respectively. (PDF) [file pdig.0000162.s009.pdf]

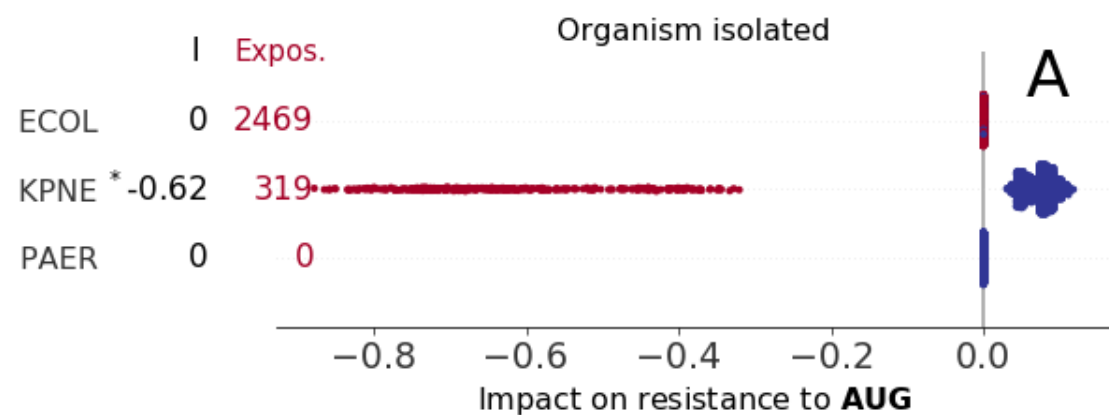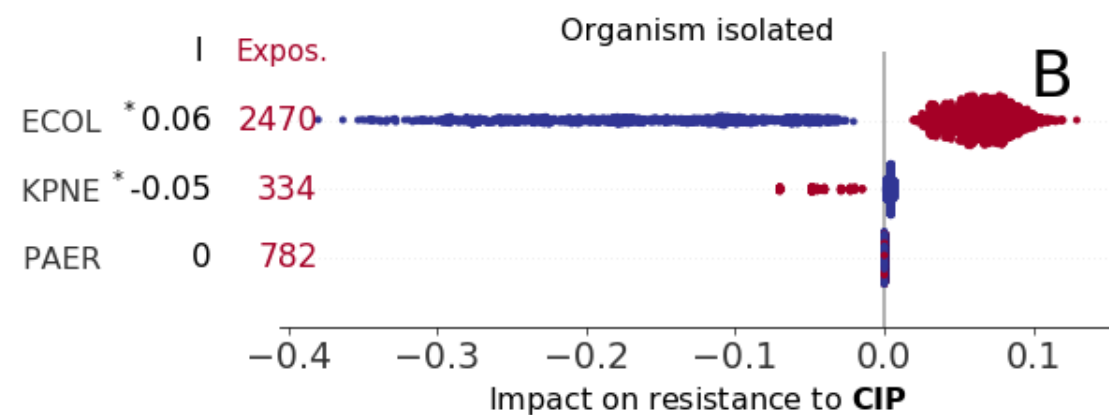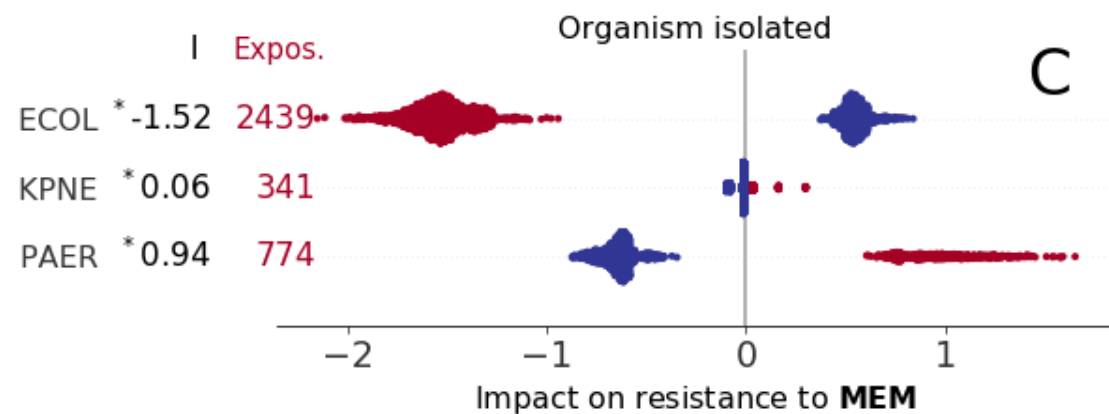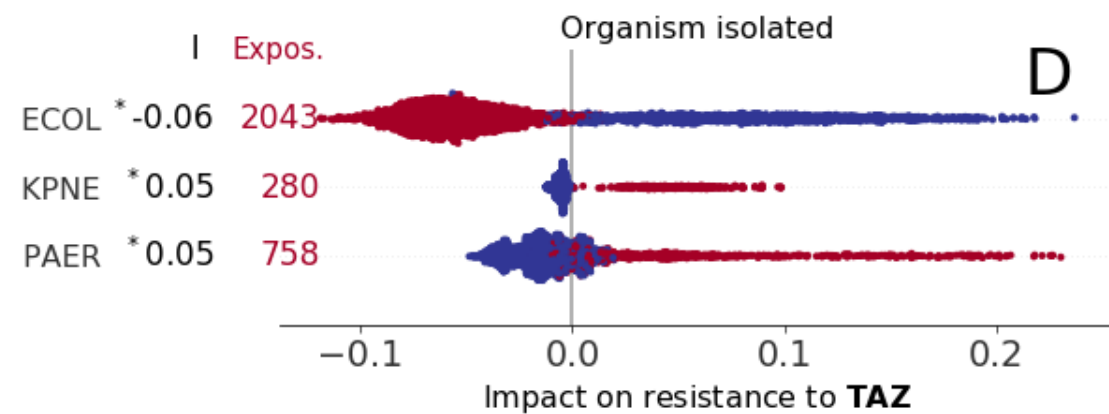

Supplement: S9 Fig — (PDF) [file pdig.0000162.s010.pdf]

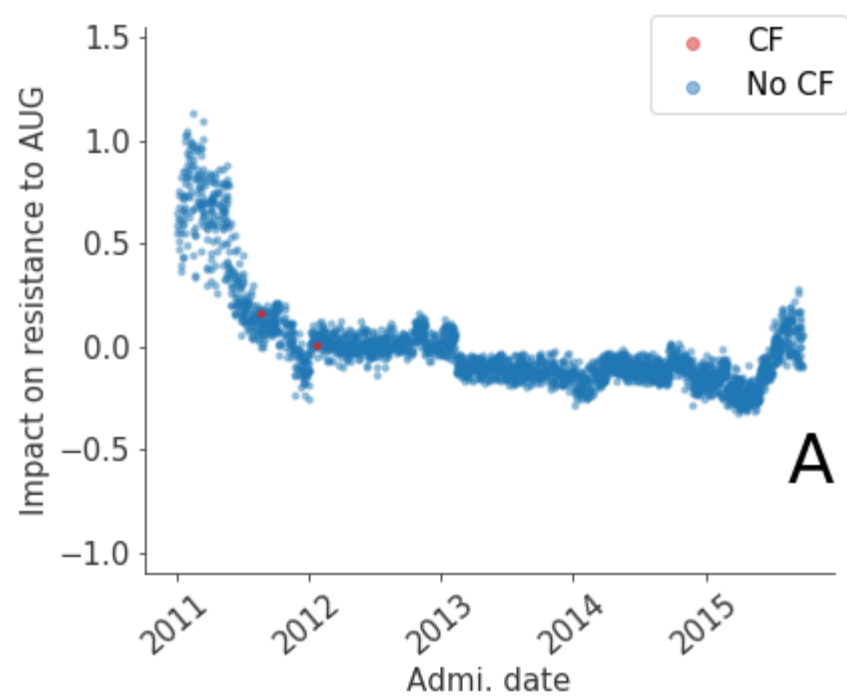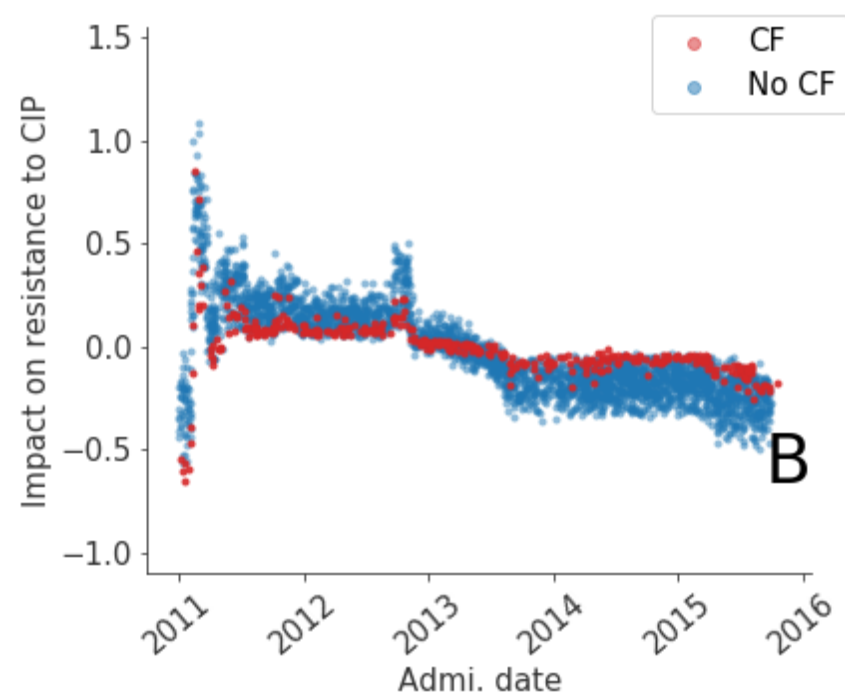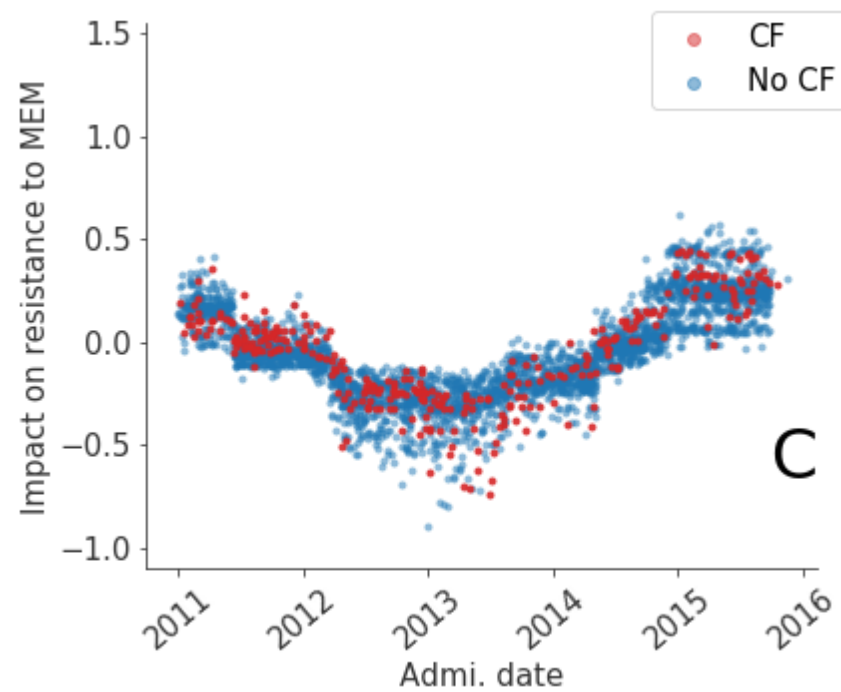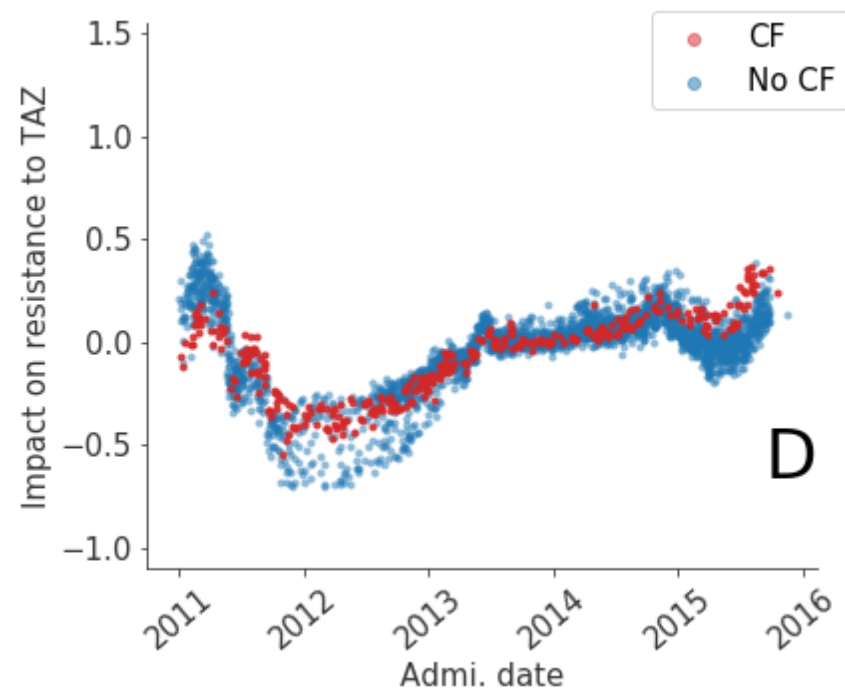

Supplement: S10 Fig — The impact varies non-linearly with the admission date. (PDF) [file pdig.0000162.s011.pdf]

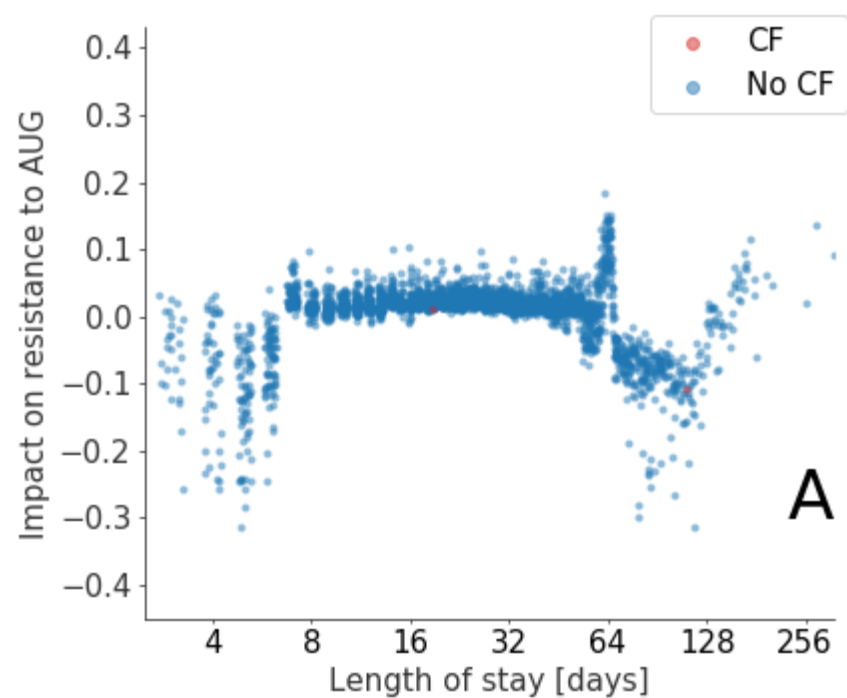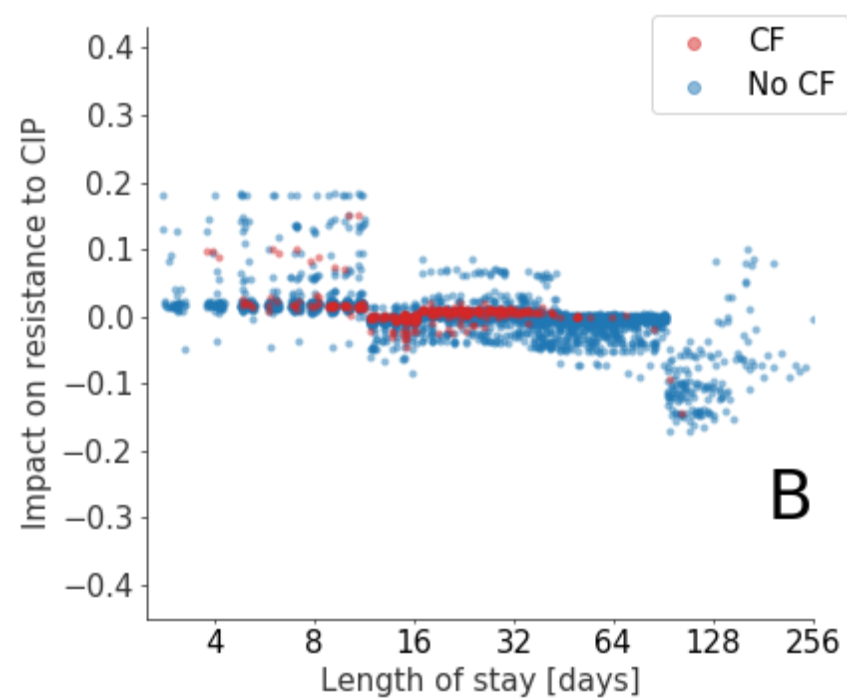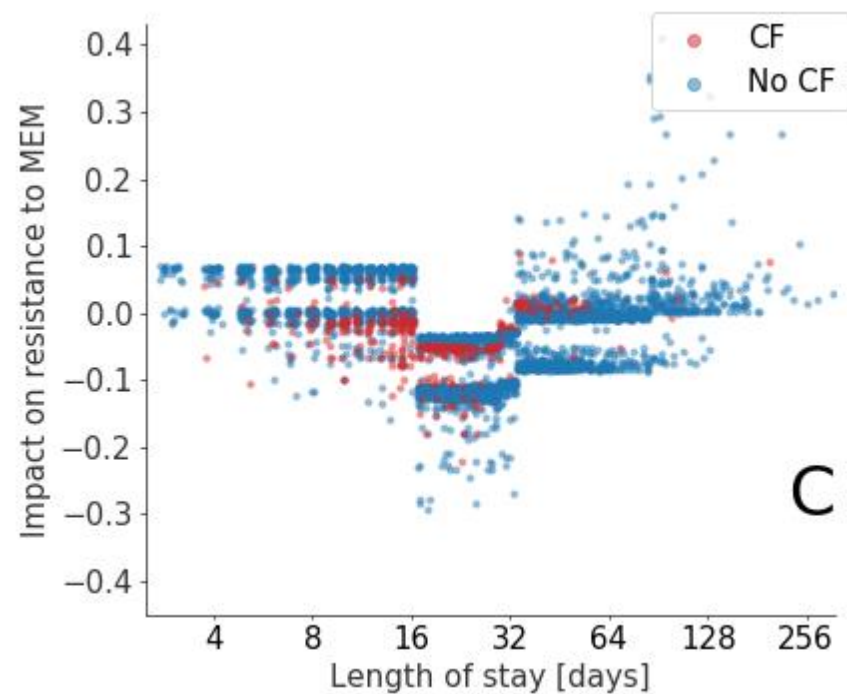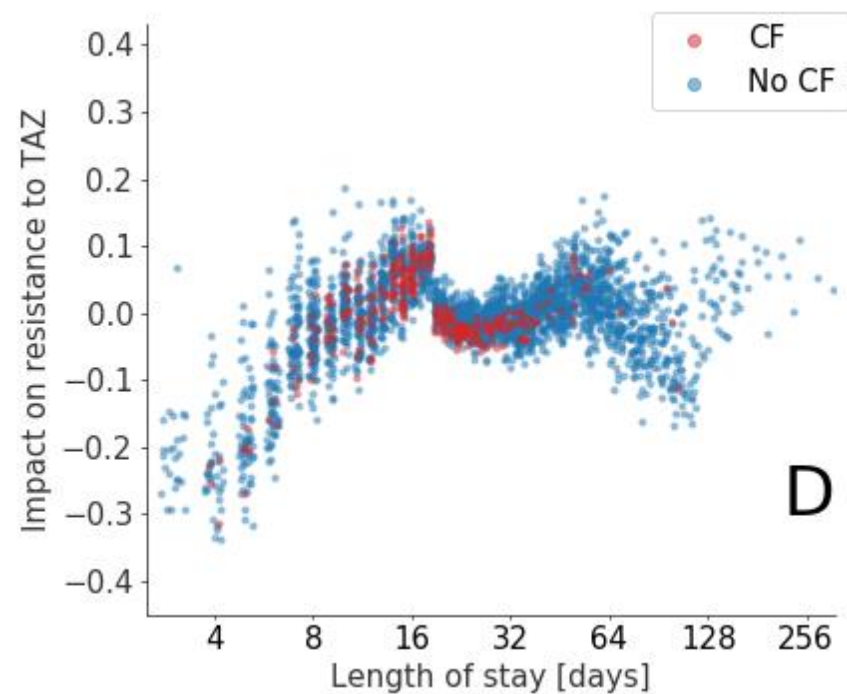

Supplement: S11 Fig — Red and blue markers corresponding to cystic fibrosis (CF) and non-CF inpatients, respectively. Impact of LOS in CF inpatients is not substantially different than in non-CF inpatiens. Horizontal axis to logarithmic scale. (PDF) [file pdig.0000162.s012.pdf]

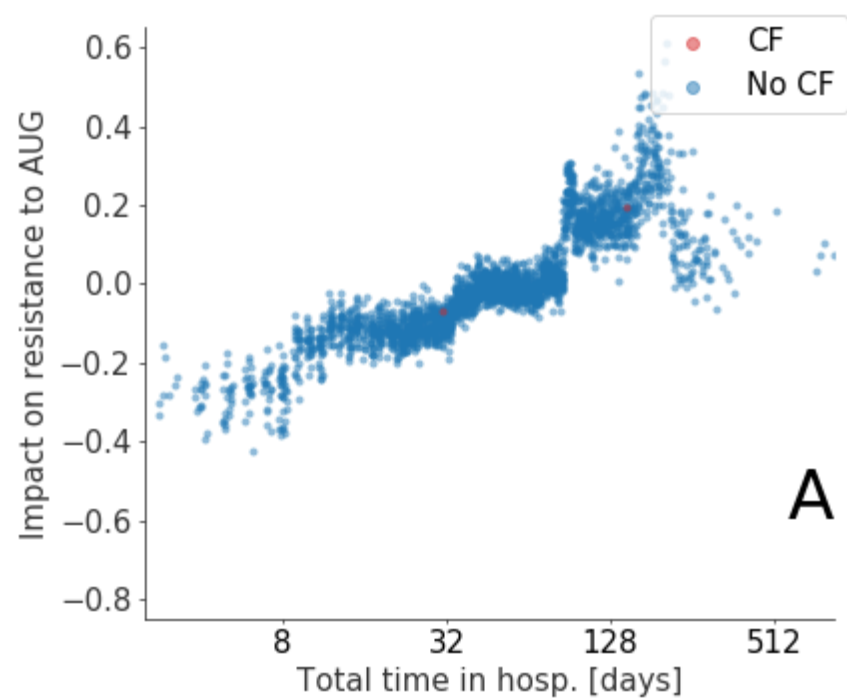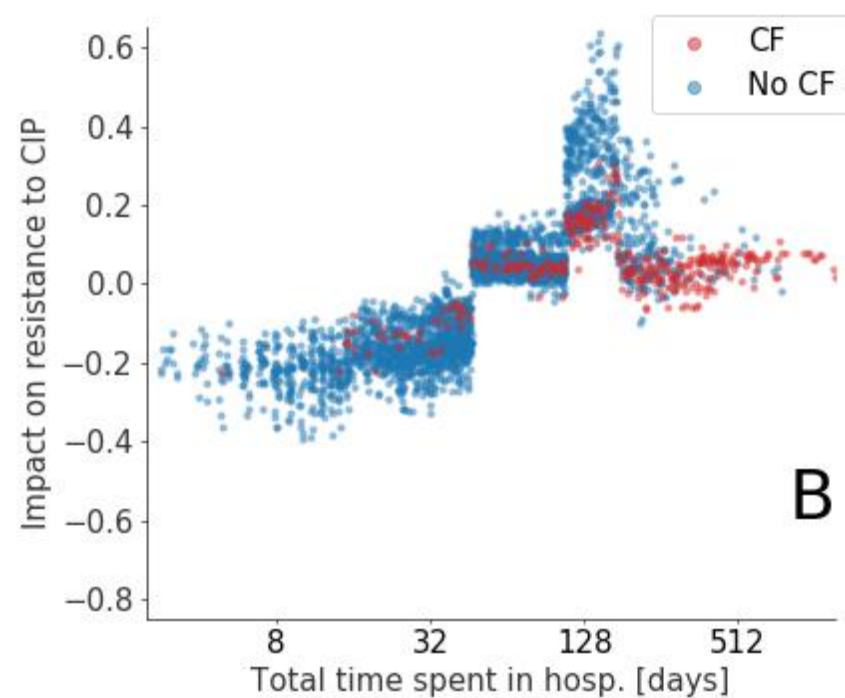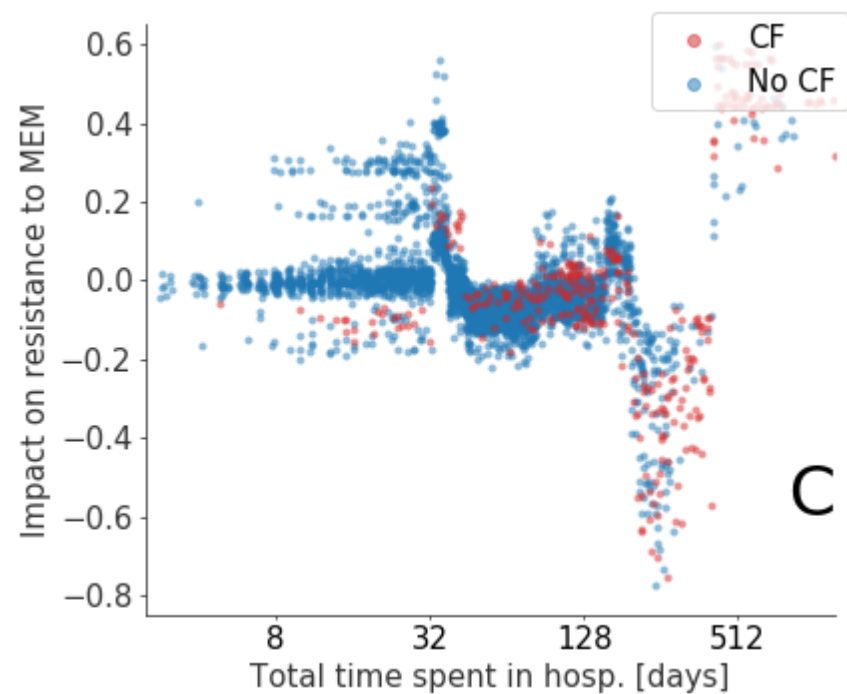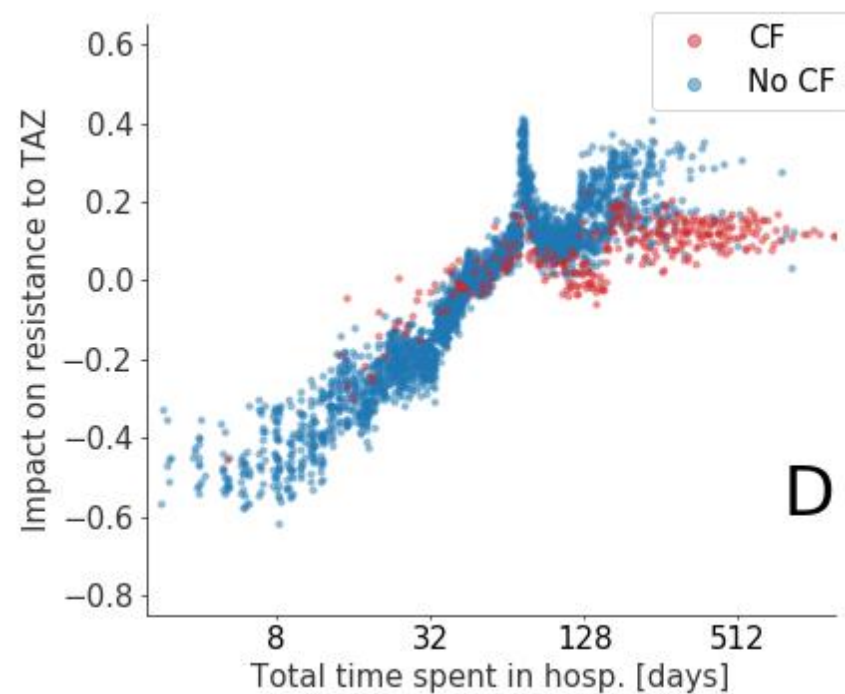

Supplement: S12 Fig — Red and blue markers corresponding to cystic fibrosis (CF) and non-CF inpatients, respectively. CF inpatients appear to spent more time in hospital than non-CF, but the impact of time in hospital for these is typically lower (B and D, CIP and TAZ respectively). Horizontal axis to logarithmic scale and jitter introduced to avoid marker overlap. (PDF) [file pdig.0000162.s013.pdf]

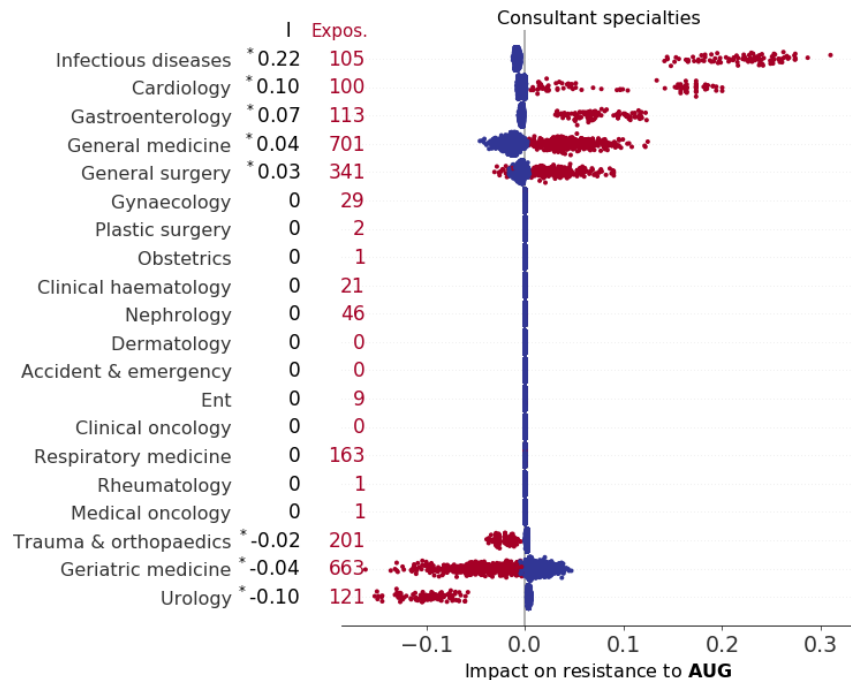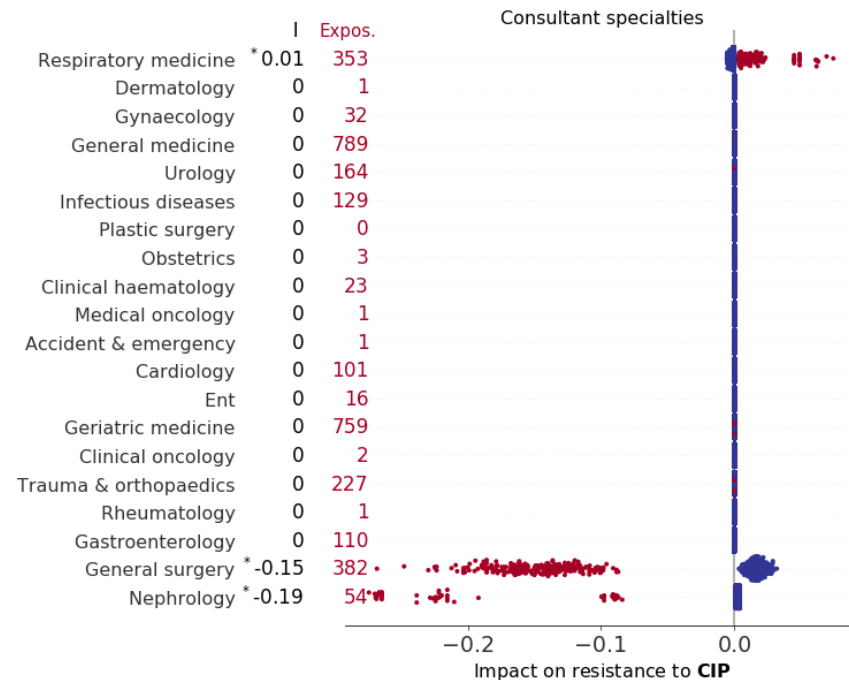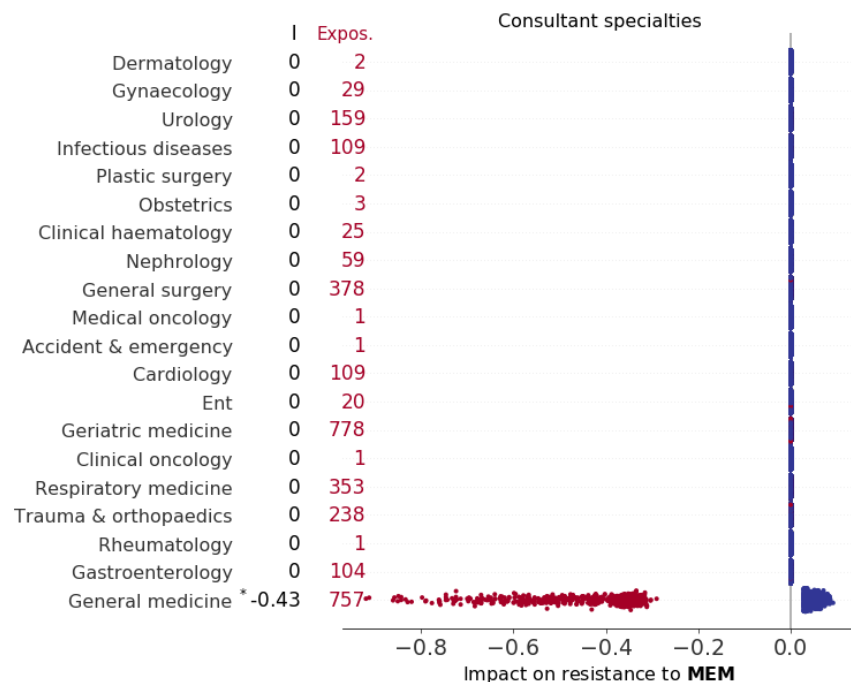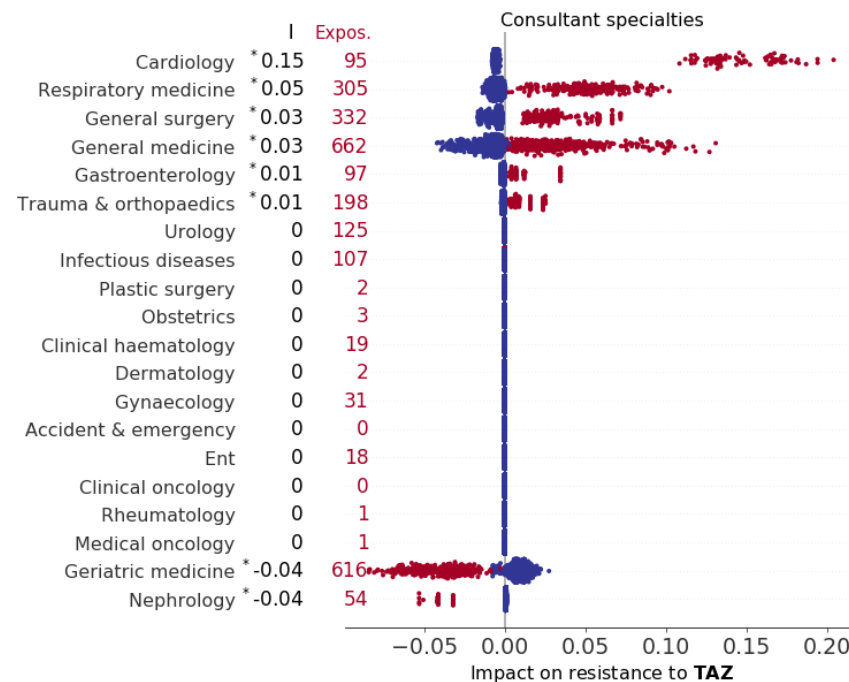

Supplement: S13 Fig — Each marker represents an admission event, horizontal coordinates representing Shapley values. Colour red indicates if the consultant responsible for the care of the patient has specialty reported on the left. The total number of consultants of each specialty is also reported on the left (Expos.). Some consultant specialties might contain information on the patients’ health and therefore also impact AI AMR prediction. According to the GBDT models, important predictors are “Infectious diseases” specialty, which appears to be associated with AMR to AUG (I = 0.22, Φ > 0.15), and “General medicine”, which appears to be negatively associated with resistance to MEM (but positively associated to resistance to AUG and TAZ). Impact of consultant specialty on resistance to broad-spectrum agents CIP and MEM is often zero. (PDF) [file pdig.0000162.s014.pdf]
