# Supplementary material for: Informing antimicrobial stewardship with explainable AI
Source: PLOS Digit Health. 2023 Jan 5;2(1):e0000162. doi: 10.1371/journal.pdig.0000162 (PMC9931350; doi:10.1371/journal.pdig.0000162)
Supplement: Attachment — Submitted filename: response_to_reviewers.docx [file pdig.0000162.s015.docx]

Dear Editor,

We thank the reviewers for providing constructive comments for improving and clarifying our manuscript. We have updated our manuscript based on their input as described in the point-by-point response below (page and line numbers refer to the file with changes highlighted). The revised manuscript also includes five additional references (Refs. [6,29-32]).

**Reviewer #1: Summary**

**The authors showed that AI-based system in the present study can predict antimicrobial resistance and reduce unmatched prescription. Moreover, the authors emphasized that antimicrobial stewardship could be possible using a Shapley explanation model. This attempt was novel and challenging, but several concerns were found throughout the study procedure.**

We thank the reviewer for recognising the novelty and the challenge of this work. The field is being developed also by raising concerns on the presentation and application of these digital technologies. We address the reviewer’s comments below.

**Major comment**

**1. Page 5, Line 77-78. The authors assumed that a prescription was done if a physician expect antimicrobial susceptible organism. On this basis, physician mismatch was determined. However, antimicrobial susceptibility is just a problem of what kind of antibiotics should be prescribed, not a problem of prescribing or not. When AI models predict the targets of antibiotic prescriptions, it is more appropriate to make decisions based on the distinction between infection and non-infection.**

The cohort used in this study was selected precisely because they had a confirmed bacterial infection (we have clarified this in the revised manuscript at page 2 line 52, “*All patients from whose blood or urine cultures bacterial pathogens Escherichia coli, Klesbiella pneumoniae, Pseudomonas aeruginosa was isolated were selected.*”). Whether an acutely admitted patient’s presentation is or is not caused by a bacterial infection is an important question but was beyond the scope of this project the purpose of which was to improve antibiotic choice for patients with confirmed bacterial infection.

We agree with the reviewer that future work should look at unselected patients and include the choice not to prescribe where that is shown to be safe. We also agree that, in practise, it is important being able to choose an antibiotic among those available, not just prescribing or not prescribing. To explain this point, we included the following text in the Discussion section (page 13, line 433):

“*The methodology can be used to help physicians reduce inappropriate use of antibiotic drugs (drugs unsuitable for a case being identified by high probability of AMR at admission) and select alternatives (drugs with lower probability of AMR, see also [15]). In particular, we recommend deploying broad-spectrum drugs (such as CIP and MEM) only when those with narrower spectrum are predicted to have high probability of resistance.*”.

We defined physician mismatch at page 5 line 92. To clarify this definition, in the revised manuscript we rephrased the text as follows:

“*A prescription made by a physician is said mismatched if at least one bacterial isolate was found to be resistant to the drug administered, during an admission event*.”

This definition has been used, e.g., in (Yelin I, et al. Nat. Med. 2019;25(7):1143), which also discussed and illustrated the importance of reducing mismatches defined in this way.

**2. The manuscript should be more concise. The content of Introduction, Methods, and Results were confused and mixed. The manuscript should be more classified each section according to their appropriate position. For example, the contents of Line 31-33 are more appropriate Methods section.**

We moved that content from the introduction into the “Materials and methods” section (page 3, line 65). We also edited and streamlined the “Results” section accordingly and moved 4 large figures from main text to SI appendix. There is always a tension between providing more details to the reader or providing a more concise narrative – we hope that the revised version of the manuscript now strikes the right balance.

**3. The description of each variable that predicted antibiotic resistance (9-19 pages) needs to be presented more concisely. Only the main results should be presented in the Result section, and I recommend that the detailed results about factors associated with AMR could be moved to the supplementary.**

As suggested by the reviewer, we shortened the manuscript: moving 4 large figures to supplementary (see also comment 2 of the same reviewer). In the revised manuscript, the part on the factors associated with antimicrobial resistance without figures is now less than 4-page long after including text at page 13, line 408, related to minor comment 3 of the same reviewer and text at page 11, line 335, as required by comment 3 of the second reviewer. It is impossible to shorten further without losing salient details.

**Minor comment**

**1. In the Methods section, please describe the statistical analysis method and the program used.**

This is explained in Methods (new text in bold) starting from line 74: “*To perform predictions, we used gradient boosted decision tree (GBDT) models with logistic objective function implemented in the XGBoost library (v0.81)* ***in Python (v3.7.1)***”

and

“*To dissect the GBDT models and explain their predictions, we perform Shapley additive explanation (SHAP) analysis of the training dataset.* ***We used an implementation specific to tree-based models, also referred to as TreeSHAP, accessible via the XGBoost and SHAP libraries*** *[20,21]*”.

**2. In the Result section, please provide baseline characteristics/demographics of the study population.**

We included a short paragraph with baseline characteristics/demographics at the end of the Results section (page 13 line 411).

**3. Page 2, Line 53. Among the data used as the input variable, consultant specialist is considered to be a variable unrelated to predicting antibiotic resistance. Why did the authors reflect this?**

One of the characteristics of machine learning methods such as the one we used in this study is that they can accommodate a large variety of input variables at negligible cost and is good practise to include them all. Moreover, a priori, we expect that consultant speciality gives some indication of the type of health condition, so it might be related to AMR – for example, a patient being cared for by urology might be expected to have more urinary tract infections.

In the Results subsection “Factors differentially associated with AMR” (page 13 line 408) we included the following sentence: “*Some consultant specialties also had an impact on AMR as illustrated in Figure S14, arguably due to the fact that they contribute some information on patient’s health conditions.*”

**4. Page 20, Line 400-421. The second paragraph of the discussion and the first part of the third paragraph overlap in the Methods section.**

We revised the Discussion section accordingly and removed overlap with Material and methods section, see also comment 2 of the same reviewer.

**Reviewer #2:**

**Applications of machine learning to the medical field, including the early expert systems, continue to flourish. However, machine learning decisions are not always intuitive and are often criticized as a black box. In this study, the authors used information on patients’ demographics, data during hospitalization, and previous drug treatment to calculate the probability of resistance and predict the likelihood of antimicrobial resistance.**

**Although there have been many studies using artificial intelligence in the medical field, none have predicted drug resistance, and I believe this is a new study. The emergence of antimicrobial resistance has always been a topic of discussion since the development of antimicrobial agents, and in recent years, appropriate use of antimicrobial agents has been demanded, especially through action plans, but inappropriate administration of antimicrobial agents is still common. I hope that this study will promote the appropriate use of antimicrobial agents.**

We thank the reviewer for their positive comments.

**I believe that the following points need to be discussed.**

**1. The use of artificial intelligence generally requires a very large amount of data. For this reason, many studies have been conducted in high-income countries with large amounts of data, and it has been pointed out that this exacerbates healthcare disparities. The use of antimicrobial agents is often high in low-income and middle-income countries, and the generalizability of this study needs to be discussed.**

We agree with the reviewer’s comment and in fact problems arising from the lack/scarcity of quality data are of very general nature. To state this point we included the following text in the Discussion section (page 18 line 466): *“[…] This broadly poses a cautionary limit to the generalisability of data-driven predictions, which cannot be easily extended to different cohorts or naively applied in situations when crucial data is missing: validation studies are necessary.*”

We also agree that antimicrobial use is often high in low- and middle-income countries and the large amount of data required for studies such as ours is not always available in these settings. Future work might include assessing whether AI-generated tools produced in one setting can be useful in another with a much more limited dataset. A discussion on how accessibility to machine learning system could drive health inequalities is important but we concluded it was beyond the scope of this paper.

**2. I believe that artificial intelligence will not replace medical professionals, but will seek a way to provide medical care in cooperation with each other. It would be good to have a discussion on the interaction with medical professionals and how this research could be used in general medical practice.**

We also believe that cooperation is key and one of the advantages of the Shapley value analysis is that it provides the physicians with a way to interpret and understand AI recommendations.

This is reported in the Discussion section (page 14 line 488, next text in bold): “*more importantly -- it can also be used by domain experts to evaluate complex automated clinical decision systems; by explaining how the AI weights factors to perform its prediction, the Shapley values can be used by physicians to determine whether AI* ***recommendations*** *are worthy of their trust.*”

and, see also response to comment 1 the first reviewer (page 13, line 433),

“*The methodology can be used to help physicians reduce inappropriate use of antibiotic drugs (drugs unsuitable for a case being identified by high probability of AMR at admission) and select alternatives (drugs with lower probability of AMR, see also [15]). In particular, we recommend deploying broad-spectrum drugs (such as CIP and MEM) only when those with narrower spectrum are predicted to have high probability of resistance.*”

We are investigating ways in which AI information can be given to physicians in real time, together with an assessment of reliability, to help inform their decision making.

**3. Although culture information and prescribed medications in previous hospitalizations are listed, unlike patient background, these data may not always be available. In the absence of such information, how accurate are the predictions in this study? For example, if these data are not available, can the accuracy be maintained by using multiple imputation to fill in missing values?**

Information on culture and prescribed medication are important for the accuracy of prediction. We demonstrated their importance by means of the Shapley value analysis and four different metrics (See table S5).

Further, quoting the text (page 11 line 325) “*Overall, past detection of a resistance or susceptible isolate was positively associated with the presence of resistance or susceptibility to the same drug, respectively (|φ| > 1 for all admissions and strong association of AUG with resistance to AUG, I = 0.58). This is by far the strongest impact on resistance result found, so past laboratory results are the single biggest indicator of current resistance (see also Table S5)*”

If data and information on culture and prescribed medication are not available, we expect that accuracy cannot be maintained. Also, multiple imputation cannot offer additional help. Indeed multiple imputation uses information already available to assign values to missing entries and the GBDT (our AI engine) already uses all the information available and actually copes well with the missing data (page 3 line 80 of revised manuscript).

By removing the variables corresponding to past culture tests, the overall accuracies of the GBDTs decrease as quantified by their ROC-AUC metrics: 0.63 (95% CI 0.60-0.65), 0.75 (95% CI 0.72-0.77), 0.97 (95% CI 0.96-0.97) and 0.59 (95% CI 0.55-0.62) for AUG, CIP, MEM and TAZ respectively, which are smaller than the ROC-AUCs achieved using all predictors. This has been included in the Results section of the revised manuscript (page 11 line 335).

We appreciate the importance of this point and our next step in this research includes an attempt to model out situations where culture information is not available. To reflect its importance we included the following text into the Discussion session (page 14 line 470, also related with comment 1):

“*Data on past culture susceptibility are crucial and in their absence, the prediction accuracy as quantified by ROC-AUC metrics decreased significantly. Yet, in practise, these data may not be always readily available for all patients in need of care. […] Our next steps also include attempts to model out situations where culture information or other variables are missing.*”

**4. Gender is assumed to have an impact on drug resistance, but it is well known that the disease trends are different between men and women, for example, women are more often affected by urinary tract infections. Could it be that past diseases are a confounding factor, resulting in the impact of gender?**

We thank the reviewer for raising this interesting point. We found that sex (our records actually reported sex rather than gender) had an impact on drug resistance (without prior assumptions) and it is not clear from the available data to what extent this is influenced by unknown confounding factors. As the number and the type of current disease are already included in the predictive model (as well as other relevant factors such as number of hospital visits), we	infer that the observed impact of sex is independent of these factors.

However, as pointed out by the reviewer there might be other unknown confounding factors, such as past diseases as the reviewer suggests. We find that sex is a significant factor that improves our model fit, however it is unclear if sex is simply acting as a reliable proxy for other more mechanistic factors. In the revised Discussed section we included the following text to comment on this aspect (page 14 line 482):

“*Sex male on average appeared to have small positive impact on resistance, arguably to be attributed to unknown confounding factors such as past comorbidities or varying adherence to the prescribed regimen, rather than to biological differences [30-32]*“

**5. Typo on line 279 "same same drug"**

We thank the reviewer for spotting the typo. We edited the text accordingly.
